# Associations Between Aromatic Compounds and Hepatorenal Biomarkers Among Coking Workers: Insights from Mediation Analysis

**DOI:** 10.3390/toxics13040298

**Published:** 2025-04-11

**Authors:** Dongming Chen, Hang Yu, Hailing Li, Guiying Li, Taicheng An

**Affiliations:** 1 Guangdong-Hong Kong-Macao Joint Laboratory for Contaminants Exposure and Health, Guangdong Key Laboratory of Environmental Catalysis and Health Risk Control, Institute of Environmental Health and Pollution Control, Guangdong University of Technology, Guangzhou 510006, China; 2112207024@mail2.gdut.edu.cn (D.C.); li_hailing777@163.com (H.L.); ligy1999@163.com (G.L.); antc99@163.com (T.A.); 2 Guangzhou Key Laboratory of Environmental Catalysis and Pollution Control, Guangdong Basic Research Center of Excellence for Ecological Security and Green Development, School of Environmental Science and Engineering, Guangdong University of Technology, Guangzhou 510006, China

**Keywords:** aromatic compounds, coking industries, Bayesian kernel machine regression, kidney function, liver function

## Abstract

Coking activities produce high concentrations of aromatic compounds (ACs) and related substances, which may have impacts on human health. However, the health effects of these substances on humans exposed to coking sites have not been fully elucidated. A total of 637 people were recruited to participate in this cross-sectional study. Using multiple linear regression and Bayesian kernel machine regression, we investigated the relationships between the urinary parent or metabolite forms of ACs (including metabolites of PAHs and their derivatives, nitrophenols, and chlorophenols) and hepatorenal biomarkers (HRBs), including total bilirubin, aspartate aminotransferase/alanine aminotransferase, serum uric acid, creatinine, albumin/globulin, and urea. The HRBs adopted in this study can effectively represent the status of human liver and kidney function. Mediation analysis was performed to investigate the possible mediating relationship between ACs and HRBs using oxidative stress markers as mediators. Our study indicated that ACs were significantly associated with increases in TBIL, AST/ALT, A/G, and UA, as well as a significant decrease in Cr. UREA showed no association with ACs among coking workers. The oxidative stress markers 8-hydroxy-2’-deoxyguanosine, 8-iso-prostaglandin-F2α, and 8-iso,15(R)-prostaglandinF2α mediated the induction of ACs on TBIL. Our results suggest that AC exposure in coking workers may be associated with adverse changes in hepatorenal biomarkers. This study highlights the significant impact of ACs from coking activities on workers’ hepatorenal biomarkers, providing crucial evidence for health risk assessment and prevention in affected populations.

## 1. Introduction

Coke production is a primary contributor to PAHs, releasing significant amounts of these pollutants into the environment [1]. China has the largest coal chemical industry in the world, employing over 400,000 people in coke oven plants [2]. Occupational exposure to aromatic compounds (ACs) in coke oven workers was significantly higher than that in non-workers [3]. Many studies have primarily focused on the coke oven occupational exposure to and health impacts of ACs. One study collected data on PAH metabolite concentrations in a population from 2013 to 2020 [4], where six out of its seven coke oven populations had ∑OH-PAHs levels exceeding 100 μg/g Cr, while only one out of fifteen general populations exceeded 65 μg/g Cr. Additionally, some researchers also detected the presence of carbazole [5], dibenzofuran [5], chlorophenols [6], and nitrophenols [6] in the wastewater of coke factories. Additionally, in the fly ash from a coking plant, these researchers detected polychlorinated dibenzofurans at a concentration of 99.6 pg/g. However, the PAH derivatives, chlorophenols, and nitrophenols of ACs are also highly toxic, but studies on occupational exposure to them and their health effects are limited.

In the liver, many ACs are metabolized primarily by cytochrome P450 enzymes [7]. When a large amount of PAHs enters the metabolic system of an organism, they may damage liver cells and eventually affect the liver [8,9]. Studies have shown that increased exposure to PAHs in humans is associated with changes in liver biomarkers [10] and with increases in liver biomarkers in pregnant women during early pregnancy [11]. Liver function tests (LFTs) are crucial diagnostic tools in essential practice for assessing liver disease [12,13]. Abnormal increases in serum bilirubin levels, such as total bilirubin (TBIL), indicate oxidative damage activity [14]. Increased aspartate aminotransferase/alanine aminotransferase (AST/ALT) ratios are linked to liver damage in hepatic disorders [15]. An elevated albumin/globulin ratio (A/G) is significantly correlated with liver fibrosis [16].

Numerous studies suggest a connection between ACs and renal function impairment [17,18,19], with higher PAH levels associated with a greater likelihood of kidney stones in the U. S. population [20]. Adaptive immune response was affected and renal fibrosis was induced in mice chronically exposed to high concentrations of benzo(a)pyrene [21]. Serum uric acid (UA), creatinine (Cr), and urea (UREA) are commonly used biomarkers for the evaluation of whether kidney function is healthy [22,23,24]. PAH derivatives, such as PAHs with alkyl groups, heteroatom-containing PAHs (HPAHs), nitro-PAHs (NPAHs), and PAHs with oxygen functional groups (OPAHs), although present at lower concentrations than unsubstituted PAHs, remain noteworthy due to their toxic potential [25,26,27]. Epidemiological studies on AC exposure and its impact on hepatorenal biomarkers (HRBs) have focused on the general population, with limited specific knowledge on AC-exposed workers.

Among the basic physiological responses of the human body, abnormalities in inflammation and oxidative stress are linked to the occurrence of many illnesses [28]. Oxidative stress biomarkers (OSBs) are frequently detected in human urine [1,29,30], including 8-hydroxy-2′-deoxyguanosine (8-OHdG) for oxidative DNA damage [31,32] and 4-hydroxy-nonenal-mercapturic acid (4-OH-NMA), 8-iso-prostaglandin-F2α (8-iso-PGF2α), 8-iso, 15 (R)-prostaglandinF2α (8-iso-15-PGF2α), and 15(R)-prostaglandinF2α (15-PGF2α) for lipid peroxidation [33,34,35]. Various ACs have been found to be associated with markers of DNA damage and peroxidative damage to lipid molecules [36,37], as well as being associated with various hepatorenal function biomarkers [10,38]. Workers in coking plants are exposed to relatively high concentrations of ACs, but studies investigating the relationship between AC concentrations and HRB levels, including the possible mediation process of OSBs, remain limited.

Specifically, this study aims to (1) explore the relationship between urinary ∑ACs and HRBs among coking industry workers; (2) explore the contributions of specific individual urinary ACs to variations in each HRB; and (3) examine how oxidative stress mediates the relationship between urinary ACs and HRBs. Our study covers a broad spectrum of ACs and OSBs currently investigated in research. Conducting this research will increase our knowledge of the effects of AC exposure on HRBs and contribute to the health monitoring of exposed workers.

## 2. Materials and Methods

### 2.1. Study Participants

This study was conducted between November and December 2020 and recruited a total of 637 adults. Questionnaires and blood and urine samples were collected from three groups: 226 workers with more than one year of work experience came from a coking plant in the largest coal-producing region in China (occupational exposure group); 163 residents lived in the areas around the coking plant (nearby residents, about 6.2 km away from the coking plant); and 248 residents lived in remote rural areas (distant rural, about 50 km away). All residents had at least one year of residence experience in their own residential area.

Fasting urine samples were gathered from the participants in the morning, placed in plastic containers, and promptly stored at −20 °C in a freezer until they were ready for analysis. The volunteers who participated in this study participated in all its necessary procedures, including completing the necessary questionnaires and providing urine and blood samples. On this basis, those with a history of major diseases (diabetes, cardiovascular, and cancer), those aged outside the 20–80 year-old range, those with positive urine protein tests, those taking traditional Chinese medicine, and those with abnormally urinary AC concentrations were excluded.

The Ethics Committee of the Guangdong University of Technology approved this study (GDUTXS20250010). All volunteers signed a written informed consent before participating in this study, which included the content of the study as well as possible risks and benefits.

### 2.2. Measurement of Serum HRBs

Fasting blood samples from all volunteers were collected in the morning at a medical institution. The serum concentrations of various HRBs, such as Cr, UREA, UA, TBIL, AST/ALT, and A/G, were analyzed using a biochemical analyzer (Roche Cobas c702 type; Roche Ltd.; Mannheim, Germany).

### 2.3. Measurement of the Urinary ACs and OSBs for Each Sample

Twenty-one ACs were detected in the urine, including ten OH-PAHs, two chlorophenols, three nitrophenols, and six PAH derivatives or metabolites of their derivatives (including 2-Naphthoic acid (2-NapCA), 4-Nitro-1-naphthol (4-OH-NNap), 5-hydroxyisoquinoline (5-OH-iQNL), 3-hydroxycarbazole (3-OH-CBZ), 2-hydroxydibenzofuran (2-OH-DBF), and 4-Chlorocatechol (4-CCT)). In addition, the concentrations of five OSBs, including one marker of oxidative DNA damage and four markers of lipid peroxidation, were analyzed. The concentrations of these urinary ACs and OSBs were determined using the recently described high-performance liquid chromatography tandem mass spectrometry method by references [39,40].

### 2.4. Assessment of Covariates

We controlled for covariates regarding sociodemographic and lifestyle factors in the statistical analyses, including age, gender, and smoking and drinking status.

### 2.5. Statistical Analysis

The normality of all environmental pollutants and human biomarkers was first assessed using the Kolmogorov–Smirnov test. As none of the data were normally distributed, medians and interquartile ranges (IQRs) were used to express the concentrations of the studied ACs, HRBs, and OSBs. For gender and drinking and smoking status, frequencies (percentages) were summarized. Kruskal–Wallis tests were used for continuous variables with a non-normal distribution and χ^2^ tests were used for categorical variables to compare their concentrations and distribution differences among the three groups. In order to make the data set present a normal distribution, it was logarithmically transformed to meet the requirements of data analysis.

Spearman correlation analysis was employed to assess the relationships between the ACs and HRBs. ACs with the same parent structure were grouped together to make the results more concise. Additionally, the Spearman analysis was performed separately for each group to examine group-specific correlation differences. In the correlation analysis, it was found that the correlation between the ACs and HRBs was stronger among coking workers. The correlation patterns of the nearby residents and remote rural population were inconsistent with those of the former group. Therefore, only the samples from the coking workers were included in the subsequent statistical analysis.

In the subsequent steps, multiple linear regression (MLR) was used to examine the linear relationship between the joint and individual urinary ACs and HRBs in coking plant workers, with age, gender, and smoking and drinking status considered as covariates. The MLR analysis included four models: Model 1, with covariates set as age and sex; Model 2, with covariates set as age, sex, and smoking status; and Model 3, with covariates set as age, sex, smoking status, and drinking status. The relationship between significantly correlated ACs and HRBs in the MLR model was calculated using restricted cubic splines, further confirming that the MLR results were robust.

Subsequently, the nonlinear effects of simultaneous and individual exposure to different ACs and human hepatorenal functions were further investigated. Bayesian kernel machine regression (BKMR) analysis was used to further explore the nonlinear and non-additive relationship between highly correlated AC exposure and HRBs [41]. HRBs were used as the dependent variable one by one and all covariates were adjusted for in each BKMR calculation. This method estimates the posterior distribution between pollutants and health indicators through a Bayesian framework to quantify uncertainty. This method combines kernel machine learning techniques to effectively capture nonlinearities and complex interactions between variables. BKMR estimates the independent effects of each pollutant by modeling the relationship between the pollutant concentrations and target variables, while also considering the joint effects of multiple pollutants. In Bayesian inference, the prior distribution is combined with the likelihood function of the data to obtain the posterior distribution, which is then used to quantify the credible interval of the model results, thereby providing uncertainty in the effects of pollutant exposure. To investigate the role of individual ACs, differences in contamination-specific exposure–response patterns and interactions were exploited by using urine contaminant levels of varying intensities while fixing other exposures at the median, 25th, and 75th percentile. Using this quantile method can better deal with collinear data, explore nonlinear relationships, and avoid the interference of extreme values on the analysis results [42].

To further verify the stability of the above model’s results, we conducted a subgroup analysis and divided the coking worker sample into male and female groups according to gender. The association between the co-exposure to ACs and HRBs within different gender groups was calculated using the BKMR model. This analysis aims to explore whether the effect of ACs on HRBs differs significantly by gender, thereby helping to confirm whether the effects of ACs are different in men and women. All statistical processes were conducted using R (version 4.2.1, Vienna, Austria) via RStudio (version 4.4.1, RStudio, Inc., Boston, MA, USA).

### 2.6. Mediation Analysis

Mediation analysis was conducted to assess whether OSBs mediate the relationship between AC exposure and HRBs. The data set used for this analysis included matched data for ACs, HRBs, and OSBs from the coking worker samples. OSBs were chosen as the mediating variable based on their potential role in DNA damage and lipid peroxidation. For each combination of ACs, OSBs, and HRBs, a separate mediation model was applied. First, a general linear regression was constructed to compute the direct correlation between the ACs and HRBs. Next, OSBs were included in the model to assess their function as intermediaries in the link between the ACs and HRBs. Through this approach, we were able to estimate both the direct effect of the ACs on HRBs and the indirect effect of OSBs in this pathway [43,44]. This analysis sought to clarify the potential role of OSBs as intermediaries in the association between AC exposure and HRBs. By interpreting the results, we gained insights into how OSBs might serve as a link in the causal pathway from AC exposure to HRBs.

## 3. Results

### 3.1. Descriptive Results

Table 1 compares the levels of serum HBRs and urinary ACs from different coking plant workers, nearby residents, and distant rural residents. Among them, the concentrations of HRBs such as UA and A/G were significantly highest in the occupational exposure group (*p* < 0.05), which were 337.5 [278, 407.33] μmol/L and 1.69% [1.53%, 1.84%]. Additionally, the TBIL levels in the workers was 15.05 [13.6, 17.3] μmol/L, significantly higher than the nearby residents (12.48 [9.64, 14.28] μmol/L) (*p* < 0.05). The total AC concentrations were significantly (*p* < 0.001) highest among the workers (Table S1, Figure S1), followed by the nearby residents, and lowest in the distant rural residents (median: 102, 57, and 30.7 μg/mL, respectively), highlighting the spread of aromatic pollutants. The distribution trends in the other HRBs in different populations did not show consistency with those of the ACs. Among the other HRBs, the median levels of Cr were 64.43, 53.25, and 84.11 μmol/L in the coking plant workers, nearby residents, and distant rural residents, respectively (*p* < 0.05). UREA was significantly lower in the workers (5.07 [4.43, 5.89] mmol/L) compared to the distant rural residents (5.5 [4.6, 6.7] mmol/L). AST/ALT were significantly highest in the residents (1.18% [0.92%, 1.4%]), followed by the workers (1.00% [0.73%, 1.16%]), and lowest in the distant rural areas (0.74% [0.61%, 0.82%]) (*p* < 0.001).

### 3.2. Associations Between Urinary ACs and HBRs in Multivariable Linear Regression

**Figure 1:**
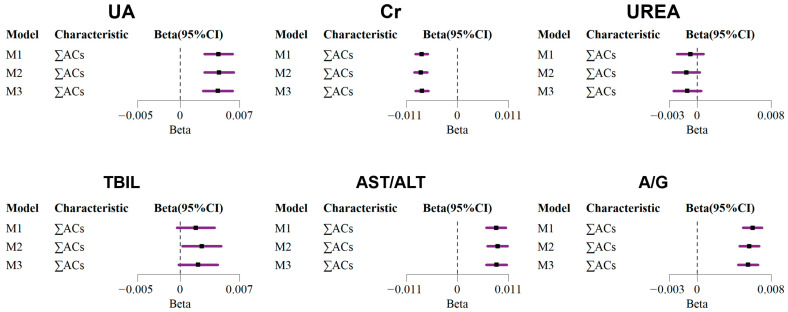
Linear regression analysis of association between natural logarithmic transformed urinary total AC concentrations and individual HRBs in coking workers. M1: model adjusted for gender (categorical) and age (continuous); M2: model adjusted for gender (categorical), age (continuous), and smoking (categorical); M3: model adjusted for gender (categorical), age (continuous), smoking (categorical), and alcohol use (categorical).

**Figure 2:**
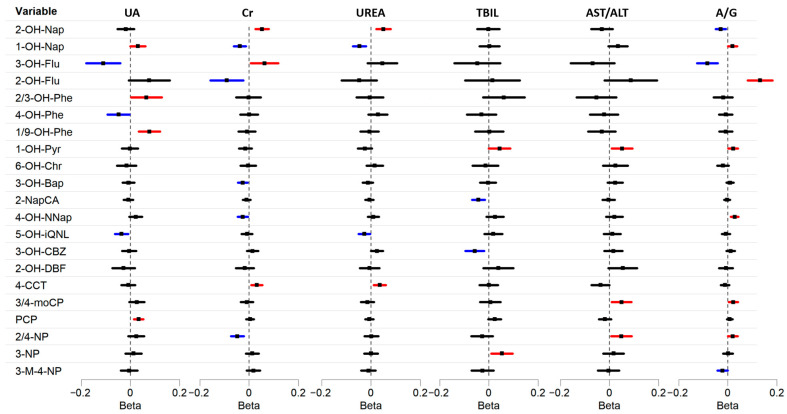
Multiple variables linear regression analysis of association between natural logarithmic transformed urinary AC concentrations and individual HRBs in coking workers. Different colors indicate the direction of the relationship: red indicates a positive relationship, blue indicates a negative relationship, and black indicates no significant relationship. Model adjusted for gender (categorical), age (continuous), smoking (categorical), and alcohol use (categorical).

In the correlation analysis, it was found that the strongest correlation was between the ACs and HRBs in the coking plant workers, while the coefficients in the nearby residents and remote rural areas were lower (Figure S2 and Table S2). Next, we performed MLR analysis to calculate the association between urinary ACs and hepatorenal function biomarkers, and the results of the models adjusted for different covariates were generally consistent. In the kidney function of the population at the coking site in the fully adjusted model, a ln-unit increase in ∑ACs was significantly associated with an increase of 0.004 (95% CI: 0.003, 0.006) in natural logarithmic UA, but a decrease of −0.008 (95% CI: −0.009, −0.006) in natural logarithmic Cr (Figure 1). Among these, a ln-unit increase in 1-OH-Nap, 2/3-OH-Phe, 1/9-OH-Phe, and PCP was significantly associated with an increase of 0.031 (95% CI: 0.000, 0.061), 0.065 (95% CI: 0.003, 0.128), 0.078 (95% CI: 0.035, 0.121), and 0.034 (95% CI: 0.015, 0.053) in natural logarithmic UA (Figure 2), respectively. Additionally, urinary 2-OH-Nap demonstrated a significant positive correlation with UREA (0.051, 95% CI: 0.021, 0.080) and Cr (0.053, 95% CI: 0.026, 0.080), while 5-OH-iQNL demonstrated a significant negative correlation with UA (−0.036, 95% CI: −0.063, −0.009) and UREA (−0.027, 95% CI: −0.050, −0.004). The specific coefficient results of the other models adjusted for different covariates (M1 and M2) are fully displayed in Table S3.

Regarding liver function (Figure 1), a ln-unit increase in ∑ACs was significantly associated with an increase of 0.0021 (95% CI: −0.0001, 0.0044) in the natural logarithm of TBIL, an increase of 0.008 (95% CI: 0.006, 0.011) in the natural logarithm of AST/ALT, and an increase of 0.006 (95% CI: 0.004, 0.007) in the natural logarithm of A/G. Among these ACs (Figure 2), the positive beta coefficient was the largest between 3-NP and TBIL (0.054, 95% CI: 0.010, 0.097), 3/4-moCP and AST/ALT (0.050, 95% CI: 0.010, 0.090), and 2-OH-Flu and A/G (0.132, 95% CI: 0.081, 0.183). A/G also had significant positive correlation with 1-OH-Nap, 1-OH-Pyr, 4-OH-NNap, 3/4-moCP, and 2/4-NP, and the coefficients were 0.019, 0.022, 0.028, 0.023, and 0.020, respectively. However, there was a negative association between some ACs and liver biomarkers, such as 3-OH-CBZ with TBIL, 4-CCT with AST/ALT, and 3-OH-Flu with A/G. To further validate these findings, we applied a restricted cubic spline analysis, which corroborated the significant associations observed in the linear regression models (Figures S3–S8), further strengthening the robustness of our results.

### 3.3. AC Exposure on Hepatorenal Biomarkers in BKMR Models

**Figure 3:**
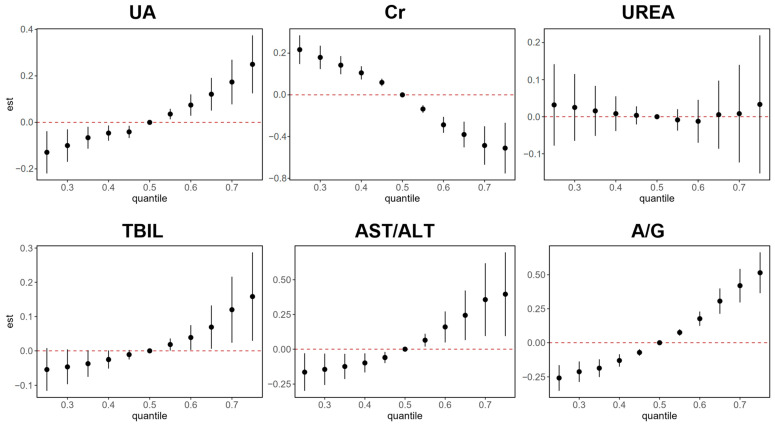
Joint association in coking workers of log-transformed urinary ACs mixture with HRBs using BKMR model’s probit extension. Overall associations (estimates and 95% CI, expressed as β probit) between pollutant mixture and liver–kidney functions when all pollutants were at particular percentiles (from 0.25 to 0.75, increments of 0.05) were compared to all pollutants at their 50th percentile. Overall association estimates were adjusted for gender (categorical), age (continuous), smoking (categorical), and alcohol use (categorical).

We used BKMR to simulate the collective impact of urinary aromatic compound concentrations on HRBs in coking plant workers (Figure 3). The analysis showed that within the combined exposure range to ACs, serum UA, TBIL, AST/ALT, and A/G levels showed statistically significant increases, ranging all percentiles. UREA levels did not show a significant trend, whereas Cr levels were negatively correlated with concurrent AC exposure.

**Figure 4:**
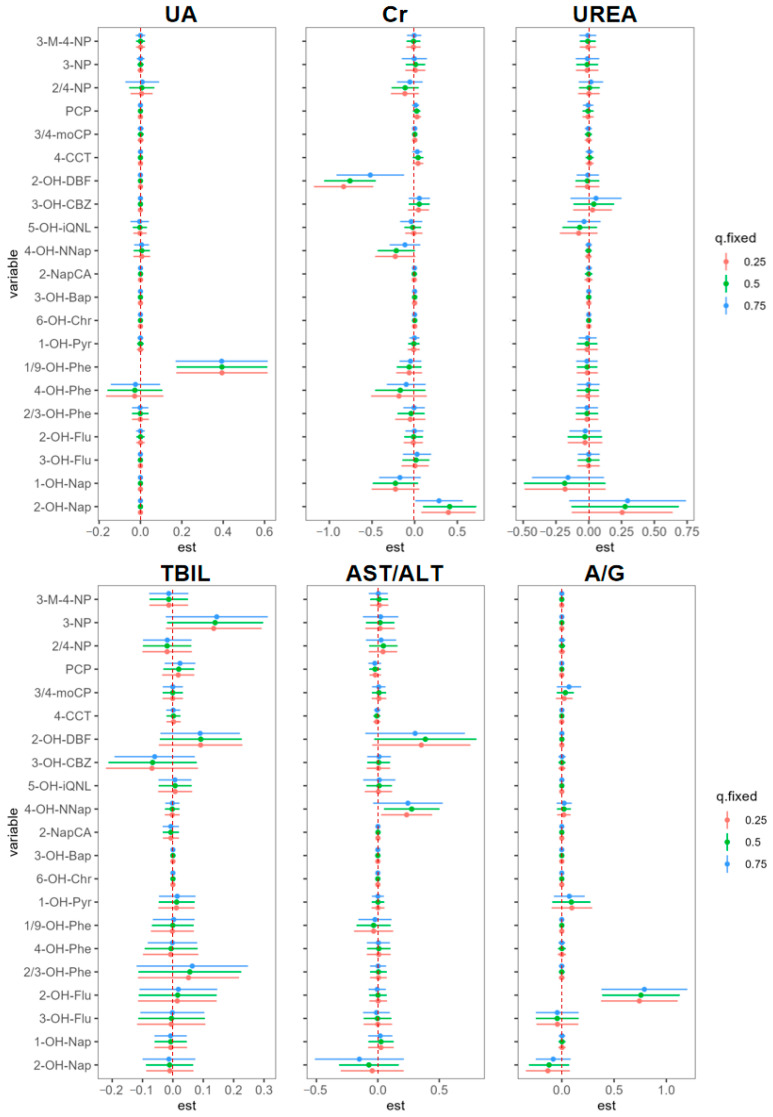
Pollutant exposure–response relationships in coking workers (estimates and 95% CI) when other AC concentrations were fixed at 25th, 50th and 75th percentiles. Models were adjusted for gender (categorical), age (continuous), smoking (categorical), and alcohol use (categorical).

The relationship between exposures to specific categories of ACs and HRBs at different exposure percentiles was further explored (Figure 4). The estimated coefficient between the natural logarithm of 1/9-OH-Phe and UA was 0.39 (95% CI: 0.17, 0.60) at all exposure percentiles (25th, 50th, and 75th) when controlling for other urinary ACs at the 50th percentile. When the urinary 2-OH-Flu concentration is controlled at the 25th percentile, an increase of one natural logarithm unit can increase the natural logarithm of A/G by 0.74 (95% CI: 0.38, 1.11). When urinary 2-OH-Flu increased to the 50th percentile, the estimated increase in the natural logarithm of A/G was 0.79 (95% CI: 0.38, 1.20). This indicates that the positive effect of 2-OH-Flu on A/G becomes stronger with increasing exposure levels. Additionally, 2-OH-Nap was positively correlated with Cr at both the 25th and 50th percentiles. When the concentration of 4-OH-NNap was controlled at the 50th percentile, it showed a significant positive correlation with AST/ALT. In contrast, urinary 2-OH-DBF was significantly negatively correlated with Cr, with the exposure–response coefficient increasing across the 25th, 50th, and 75th percentiles. Individual ACs had no significant relationship with TBIL and UREA, regardless of whether their concentration quantiles were controlled at any percentiles. The calculation results of the posterior inclusion probabilities (Table S4) verified the estimation results of BKMR.

### 3.4. Mediation Analysis Results

**Figure 5:**
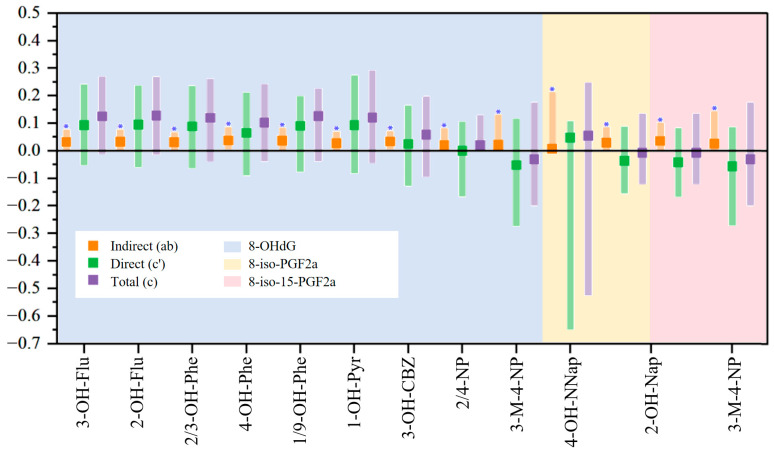
Depicts the mediation analysis of OSBs in the relationship between AC exposure and TBIL in coking workers. Indirect (ab): average causal mediation effect; Direct (c’): average direct effect; Total (c): total effect. Models were adjusted for gender (categorical), age (continuous), smoking (categorical), and alcohol use (categorical).

Oxidative stress damage may play a role in the process of environmental pollutants affecting human health, and we did not observe a significant association between individual ACs and the liver function biomarker TBIL in the analysis results of BKMR. Therefore, we used five OSBs for mediation analysis to confirm their possible average causal mediating role of individual ACs for HRB changes. 8-OHdG was found to mediate 23.14–58.62% of the AC-related increase in TBIL (*p* < 0.05), including exposures to six OH-PAHs and a metabolite of a PAH derivative (Figure 5). Additionally, 8-iso-PGF2α mediated 12.96% of the elevated TBIL associated with 4-OH-NNap (*p* < 0.05). 8-iso-15-PGF2α only played a mediating role in reducing TBIL in the negative correlation between two ACs and TBIL. In addition, no significant mediating effect of OSBs was found between other ACs and HRBs. Although the direct and total effects remained non-significant (Table S5), these findings suggest that the effects of ACs on TBIL are partly mediated by oxidative stress insults, such as DNA damage and lipid inflammation.

### 3.5. Subgroup Analysis

We performed subgroup analysis to compare the relationship between urinary ACs and HRBs calculated by BKMR to confirm whether there were different performances in different sex groups. The dose–response relationships between the HRBs and total urine ACs in men and women were similar in the overall population, except for in TBIL (Figures S9 and S10). The BKMR dose–response curves for TBIL and the urinary ACs in the general population of coking plant workers and male workers were consistently positively correlated, but the relationship in the female population was different. The imbalance in sample size between the males and females may have led to variations in these dose–response patterns, with the larger sample size in males potentially obscuring the female response. These discrepancies could be attributed to differences in exposure levels, biological variations, and other potential factors [10,45].

## 4. Discussion

In this study of coking sites, we observed a significant correlation between urinary ACs and some HRBs as a result of mathematical statistics. Two OSBs were observed to mediate the relationship between ACs and HRBs in a mediation analysis. Specifically, ∑ACs was significantly associated with increases in TBIL, AST/ALT, A/G, and UA, as well as a significant decrease in Cr, and UREA showed no association. Oxidative stress played a mediating role in the increase in TBIL caused by ACs in the coking industry population.

### 4.1. Association of Urinary AC Metabolites with Kidney Biomarkers

The concentration distribution differences in UA across different populations were consistent with the distribution trends in ACs, whereas those of Cr and UREA were not. The consistent positive association between 1/9-OH-Phe and UA was confirmed through MLR and BKMR analyses, whereas neither Cr nor UREA exhibited a consistent and robust positive correlation with the ACs. This suggests that ACs primarily affect a portion of kidney function represented by UA, while Cr and UREA may be predominantly influenced by other factors.

Limited research has explored the impact of AC exposure on the risk of hyperuricemia [40]. A study conducted at a coking site in Southern China revealed a strong association between ΣOH-Phe and an increased risk of hyperuricemia, which supports our findings. Additionally, mouse experiments have suggested that PAHs may impair kidney function, leading to elevated UA levels with increased PAH exposure [46]. Under conditions of mixed AC exposure, we observed a negative correlation with Cr, primarily influenced by 2-OH-DBF. However, our model also indicated a significant positive correlation between 2-OH-Nap and Cr. This highlights the intricate nature of the relationship between ACs and Cr, necessitating further comprehensive investigation. Studies of petrochemical complexes have indicated that higher AC exposure levels are linked to adverse kidney function, although the specific kidney health indicators studied differed from those in our research [47]. A study reported a positive correlation between PAH exposure and Cr in southern China; specifically, exposure to the 75th and 95th percentiles of 1-OH-Nap increased Cr by 8.432 (1.713, 15.151) μmol/L [48]. While our study’s overall findings on ACs differ from these results, both studies underscore the positive association between naphthalene exposure and Cr. In contrast, a longitudinal panel study conducted in central China found that Chrysene, Benzo(a)anthracene, Benzo(a)pyrene, and 2,3-(o-Phenylene)pyrene in PM2.5 were positively correlated with UREA [49]. However, there was no association between UREA and urinary AC concentrations in our study population. Nevertheless, our research showed that exposure to ACs is associated with impaired kidney function, particularly manifesting in elevated UA levels.

Although several studies have proposed that ACs impact kidney function through oxidative stress pathways [38,50], our mediation analysis did not provide evidence supporting OSBs as mediators between ACs and kidney function. This may be due to the AC exposure levels in coking workers and nearby residents, which are higher than those in the other studied populations, and these elevated concentrations of ACs may have directly impaired kidney function.

### 4.2. Association of Urinary AC Metabolites with Liver Biomarkers

The increase in TBIL and AST/ALT levels signifies liver damage [14,15], whereas an elevated A/G ratio is linked to pulmonary fibrosis [16]. Studies conducted in pregnant women and adolescents suggest that AC exposure can affect liver function indicators [11,51]. In our coking site, we observed a significant positive correlation between mixed AC exposure and TBIL, AST/ALT, and A/G. Numerous studies exploring the impact of specific ACs on liver function indicators such as TBIL, AST, and ALT suggest that ACs can detrimentally affect liver function [52], which supports our findings. However, research examining the association between ACs and A/G is scarce. A study focusing on psychiatric patients found that B(a)A, B(b)F, B(K)F, and DB(a,h)A were positively correlated with A/G, whereas fluorene did not show a significant relationship [53]. In our study, the positive correlation between 2-OH-Flu and A/G was consistently observed across multiple statistical analyses, suggesting that the relationship between the total AC concentration and A/G is likely dominated by the correlation between 2-OH-Flu and A/G, indicating a potential risk of liver damage. Our study also found that the relationship between ACs and TBIL in different gender subgroups was inconsistent in males and females, which is similar to the results of previous studies [10]. This indicates that it is necessary to further expand the sample size to study whether the difference in the results was caused by an imbalance of samples, or to further study the different mechanisms affecting TBIL in ACs due to gender differences.

Our study on the coking site population found that 8-OHdG mediates the relationship between ACs and TBIL, which is consistent with previous studies. Multiple studies suggest that oxidative stress plays intermediary roles in the connection between specific ACs and liver biomarkers [10,54,55]. OH-PAHs in the human body are associated with oxidative stress [56]. Mono-nitrophenols induced oxidative stress in human lung cells in a vitro study [57]. An increase in oxidative stress biomarkers may lead to an upregulation of liver enzymes, potentially contributing to hepatotoxicity [58]. This effect could be mediated by the activation of specific receptors, such as constructive androstane receptors (CARs), which regulate the expression of enzymes like CYP2B1 and CYP2B2 [59]. Studies focusing on adolescents also suggest that inflammation may mediate the toxic effects of PAHs on liver function [51]. Studies on 4-NP in zebrafish have shown that it can induce oxidative stress and induce liver abnormalities [60].

A random sampling study in Guangzhou also found a link between PAH exposure and elevated liver function indicators [10]. Its authors found that the relationship between TBIL and PAHs was not statistically significant, and WBC could mediate the association between substances like OH-Nap and OH-Flu with GGT and ALP. In contrast, 8-OHdG was identified as a negative mediator between 3-OH-Phe and TBIL. In our study, AC exposure levels were higher, urinary ACs in the coking workers were significantly positively correlated with TBIL, and oxidative stress biomarkers mediated this relationship. This suggests that the higher AC exposure in the coking population has a more pronounced impact on liver function. The inclusion of a broader range of ACs in our study makes the findings more comprehensive. This study contributes robust empirical evidence supporting the notion that concurrent exposure to ACs could significantly impact serum biomarkers associated with liver and kidney function. While elevations in HRBs within normal ranges may not signify notable pathological implications [61], our findings are crucial for public health efforts, particularly in identifying risk factors linked to prolonged AC exposure among populations residing near coking plants.

### 4.3. Limitations and Strengths

Innovatively, this study of the relationship between HRBs and urinary ACs in coking plant workers and residents near coking plants is the most comprehensive analysis to date. Moreover, examining the cumulative effects of AC exposure provides a deeper understanding and greater value compared to focusing on the impacts of each chemical separately [62]. However, our study has several acknowledged limitations. Firstly, the observational nature of our research limits its ability to infer cause-and-effect links between AC exposure and the outcomes we observed. The mediating effect of oxidative stress in this study is not high. ACs may also contribute to the occurrence of oxidative stress by affecting liver function. While our findings suggest that AC exposure may detrimentally influence HRBs, only prospective clinical trials can definitively assess the possibility of developing serious hepatorenal diseases over extended periods [63] and clarify the outcome direction of the mediating effect. Additionally, the relationship between TBIL and the ACs in the female groups was not consistent. The imbalance in the sample sizes between the males and females may have resulted in variations in the dose–response pattern, with the larger sample size in males potentially masking the response in females. Biodiversity, different exposure levels to ACs, and other potential factors may have contributed to this inconsistency. Finally, despite our efforts to include as many relevant ACs as possible in this study, the potential contributions of other unmeasured ACs to alterations in HRBs cannot be disregarded. Notwithstanding these limitations, our study underscores the necessity for further research into the impact of AC exposure on HRBs within affected populations.

## 5. Conclusions

In conclusion, this study identified the association between simultaneous and individual exposure to ACs and HRBs in coking workers, and the mediating role of oxidative stress was further emphasized. The serum liver biomarkers TBIL, AST/ALT, and A/G were elevated, as was UA for the kidney biomarker, and oxidative stress mediated AC-related TBIL elevation. Further research is essential to validate these initial findings and to evaluate the hepatorenal toxicity associated with exposure to ACs in the coking industry.

## Figures and Tables

**Table 1:** Study population characteristics.

Characteristic	Worker (*n* = 226)	Resident (*n* = 163)	Control (*n* = 248)
Age	45.88 [35.71, 51.15]	57.38 [49.7, 64.59]	51 [39.25, 58]
Sex (%)			
Male	174 (77)	46 (28.2)	77 (31)
Female	52 (23)	117 (71.8)	171 (69)
Smoking Status (%)			
Current smokers	87 (38.5)	125 (76.7)	190 (76.6)
Other	139 (61.5)	38 (23.3)	58 (23.4)
Drinking Status (%)			
Current drinkers	102 (45.1)	133 (81.6)	219 (88.3)
Other	124 (54.9)	30 (18.4)	29 (11.7)
Kidney function biomarkers			
Cr (μmol/L)	64.43 [56.78, 72.15]	53.25 [48.09, 60.14]	84.11 [73.37, 93.35]
UREA (mmol/L)	5.07 [4.43, 5.89]	4.91 [4.19, 5.73]	5.5 [4.6, 6.7]
UA (μmol/L)	337.5 [278, 407.33]	275 [221.75, 324.25]	260.75 [213.73, 311.53]
Liver function biomarkers			
TBIL (μmol/L)	15.05 [11, 13.6, 17.3]	12.48 [9.64, 14.28]	13.14 [9, 15]
AST/ALT (%)	1 [0.73, 1.16]	1.18 [0.92, 1.4]	0.74 [0.61, 0.82]
A/G (%)	1.69 [1.53, 1.84]	1.46 [1.35, 1.59]	1.23 [1.11, 1.31]
Urine AC concentration (ng/mL)			
2-OH-Nap	20.16 [8.52, 46.3]	6.97 [3.33, 13.72]	4.34 [2.06, 9.98]
1-OH-Nap	18.53 [8.47, 33.97]	7.39 [3.13, 15.19]	2.6 [1.53, 5.92]
3-OH-Flu	3.89 [2.39, 8.32]	1.23 [0.74, 2.02]	0.7 [0.46, 1.24]
2-OH-Flu	8.41 [4.65, 16.78]	2.11 [1.27, 3.74]	0.87 [0.52, 1.75]
2/3-OH-Phe	1.16 [0.67, 2.59]	0.52 [0.35, 0.9]	0.23 [0.15, 0.4]
4-OH-Phe	1.31 [0.77, 2.55]	0.63 [0.38, 1.17]	0.32 [0.19, 0.54]
1/9-OH-Phe	0.27 [0.15, 0.48]	0.11 [0.07, 0.21]	0.07 [0.03, 0.11]
1-OH-Pyr	4.56 [2.12, 11.9]	0.9 [0.48, 1.55]	0.39 [0.21, 0.73]
6-OH-Chr	0 [0, 0.03]	0 [0, 0.01]	0 [0, 0]
3-OH-Bap	0.01 [0, 0.12]	0.01 [0, 0.07]	0 [0, 0.01]
2-NapCA	1.43 [0.65, 3.77]	0.66 [0.26, 2.34]	0.3 [0.13, 0.78]
4-OH-NNap	0.08 [0.05, 0.15]	0.06 [0.03, 0.1]	0.02 [0.01, 0.04]
5-OH-iQNL	7.91 [5.62, 12.37]	6.94 [3.53, 12.84]	5.37 [2.94, 9.67]
3-OH-CBZ	0.69 [0.36, 1.54]	0.2 [0.11, 0.4]	0.12 [0.06, 0.31]
2-OH-DBF	9.94 [5.78, 18.72]	2.6 [1.67, 5.01]	1.32 [0.81, 2.35]
4-CCT	0.62 [0.39, 1.05]	0.52 [0.3, 0.83]	0.44 [0.28, 0.81]
3/4-moCP	0.05 [0.03, 0.09]	0.03 [0.02, 0.05]	0.03 [0, 0.04]
PCP	0.09 [0.04, 0.28]	0.02 [0.01, 0.08]	0.03 [0, 0.07]
2/4-NP	1.63 [1, 2.29]	1.39 [0.82, 2.3]	0.68 [0.39, 1.14]
3-NP	0.05 [0.03, 0.07]	0.03 [0.02, 0.05]	0.02 [0, 0.04]
3-M-4-NP	0.12 [0.07, 0.21]	0.1 [0.06, 0.23]	0.04 [0, 0.09]
Total	102 [21.2–1150]	57.0 [11.5–523]	30.7 [7.72–798]
OSB concentration (ng/mL)			
8-OHdG	0.81 [0.54, 1.08]	---	---
4-OH-NMA	45.26 [11.03, 129.23]	---	---
8-iso-PGF2α	0.61 [0.46, 0.9]	---	---
8-iso-15-PGF2α	0.35 [0.24, 0.58]	---	---
15-PGF2α	1.13 [0.79, 1.63]	---	---

Other, former smokers and nonsmokers or former drinkers and nondrinkers. The parameter of the median and IQR was set for non-normal distributed graded data, *n* (%), for quantal data. The comparison of concentrations between the environmental media for different groups was conducted using the Kruskal–Wallis method, and multiple comparisons were carried out in pairs via the Bonferroni method. ---, the data for this part are unavailable.

## Data Availability

The data presented in this study are available on request from the corresponding author since they involve the personal privacy of the volunteers.

## Supplementary Materials

The following supporting information can be downloaded at https://www.mdpi.com/article/10.3390/toxics13040298/s1, Figure S1: Level of kidney function and concentrations of urinary pollutants in different regions; Figure S2: Spearman correlation between urinary pollutants and kidney function tests; Figure S3: Adjusted restricted cubic splines of the associations between 1-OH-Nap, 3-OH-Flu, 4-OH-Phe, 1/9-OH-Phe, 2/3-OH-Phe, 5-OH-iQNL, PCP, and UA; Figure S4: Adjusted restricted cubic splines of the associations between 1-OH-Nap, 2-OH-Nap, 2-OH-Flu, 3-OH-Flu, 4-OH-NNap, 4-CCT, 3-OH-Bap, 2/4-NP, and Cr; Figure S5: Adjusted restricted cubic splines of the associations between 1-OH-Nap, 2-OH-Nap, 5-OH-iQNL, 4-CCT, and UREA; Figure S6: Adjusted restricted cubic splines of the associations between 1-OH-Pyr, 2-NapCA, 3-OH-CBZ, 3-NP, and TBIL; Figure S7: Adjusted restricted cubic splines of the associations between 1-OH-Pyr, 3/4-moCP, 2/4-NP, and AST/ALT; Figure S8: Adjusted restricted cubic splines of the associations between 1-OH-Nap, 2-OH-Nap, 2-OH-Flu, 1-OH-Pyr, 4-OH-NNap, 2/4-NP, 3-M-4-NP, 3/4-moCP, and A/G; Figure S9: Joint effects of urinary OH-PAHs on kidney function biomarker strategy by gender; Figure S10: Joint effects of urinary OH-PAHs on liver function biomarker strategy by gender; Table S1: Study population characteristics; Table S2: Spearman correlation coefficients among urinary ACs in coking worker participants; Table S3: Multivariable linear regression analysis of AC and HRB coefficients (β, 95%CI); Table S4: Estimated posterior inclusion probabilities (PIPs) for HRBs through Bayesian kernel machine regression (BKMR); Table S5: The mediation effect coefficients of OSBs along with their 95% confidence intervals.

## Abbreviations

The following abbreviations are used in this manuscript:

ACs	Aromatic compounds
HRBs	Hepatorenal biomarkers
OSBs	Oxidative stress biomarkers
MLR	Multiple linear regression
BKMR	Bayesian kernel machine regression
TBIL	Serum total bilirubin
AST/ALT	Serum aspartate aminotransferase/alanine aminotransferase
A/G	Serum albumin/globulin
UA	Serum uric acid
Cr	Serum creatinine
UREA	Serum urea
2-OH-Nap	2-hydroxynaphthalene
1-OH-Nap	1-hydroxynaphthalene
3-OH-Flu	3-hydroxyfluorene
2-OH-Flu	2-hydroxyfluorene
2/3-OH-Phe	2/3-hydroxyphenanthrene
4-OH-Phe	4-hydroxyphenanthrene
1/9-OH-Phe	1/9-hydroxyphenanthrene
1-OH-Pyr	1-Hydroxy-Pyrene
6-OH-Chr	6-Hydroxychrysene
3-OH-Bap	3-Hydroxybenzo(A)Pyrene
2-NapCA	2-Naphthoic acid
4-OH-NNap	4-Nitro-1-naphthol
5-OH-iQNL	5-hydroxyisoquinoline
3-OH-CBZ	3-hydroxycarbazole
2-OH-DBF	2-hydroxydibenzofuran
4-CCT	4-Chlorocatechol
3/4-moCP	3/4-monochlorophenol
PCP	Pentachlorophenol
2/4-NP	2/4-Nitrophenol
3-NP	3-Nitrophenol
3-M-4-NP	3-methyl-4-nitrophenol
8-OHdG	8-hydroxy-2’-deoxyguanosine
8-iso-PGF2α	8-iso-prostaglandin-F2α
8-iso-15-PGF2α	8-iso,15(R)-prostaglandinF2α
15-PGF2α	15(R)-prostaglandinF2α
4-OH-NMA	4-hydroxy-nonenal-mercapturic acid
