# Supplementary material for: Associations Between Aromatic Compounds and Hepatorenal Biomarkers Among Coking Workers: Insights from Mediation Analysis
Source: Toxics. 2025 Apr 11;13(4):298. doi: 10.3390/toxics13040298 (PMC12031308; doi:10.3390/toxics13040298)
Supplement: Supplementary file 1 [file toxics-13-00298-s001.zip › toxics-3539721-supplementary.pdf]

Supplementary material (SI)

Associations Between Aromatic Compounds and Hepatorenal Biomarkers among Coking workers: Insights from Mediation Analysis

Dongming Chen[1, 2], Hang Yu[1, 2, *], Hailing L[1, 2], Guiying L[1, 2], Taicheng An[1, 2]

[1] *Guangdong-Hong Kong-Macao Joint Laboratory for Contaminants Exposure and Health, Guangdong Key Laboratory of Environmental Catalysis and Health Risk Control, Institute of Environmental Health and Pollution Control, Guangdong University of Technology, Guangzhou 510006, China;*

[2] *Guangzhou Key Laboratory of Environmental Catalysis and Pollution Control, Guangdong Basic Research Center of Excellence for Ecological Security and Green Development, School of Environmental Science and Engineering, Guangdong University of Technology, Guangzhou 510006, China.*

***Corresponding Author: A/Prof. Hang Yu,** E-mail: hangxyu@163.com

Table of Contents

**Fig. S1 Level of kidney function and concentrations of urinary pollutants in different regions.**	**3**
**Fig. S2 Spearman correlation between urinary pollutants and kidney function tests.**	**3**
**Fig. S3 Adjusted restricted cubic splines of the associations between 1-OH-Nap, 3-OH-Flu, 4-OH-Phe, 1/9-OH-Phe, 2/3-OH-Phe, 5-OH-iQNL, PCP and UA.**	**4**
**Fig. S4 Adjusted restricted cubic splines of the associations between 1-OH-Nap, 2-OH-Nap, 2-OH-Flu, 3-OH-Flu, 4-OH-NNap, 4-CCT, 3-OH-Bap, 2/4-NP and Cr.**	**4**
**Fig. S5 Adjusted restricted cubic splines of the associations between 1-OH-Nap, 2-OH-Nap, 5-OH-iQNL, 4-CCT and UREA.**	**5**
**Fig. S6 Adjusted restricted cubic splines of the associations between 1-OH-Pyr, 2-NapCA, 3-OH-CBZ , 3-NP and TBIL.**	**5**
**Fig. S7 Adjusted restricted cubic splines of the associations between 1-OH-Pyr, 3/4-moCP, 2/4-NP and AST/ALT.**	**6**
**Fig. S8 Adjusted restricted cubic splines of the associations between 1-OH-Nap, 2-OH-Nap, 2-OH-Flu, 1-OH-Pyr, 4-OH-NNap, 2/4-NP, 3-M-4-NP, 3/4-moCP and A/G.**	**6**
**Fig. S9 Joint effects of urinary OH-PAHs on kidney function biomarkers strategy by gender.**	**7**
**Fig. S10 Joint effects of urinary OH-PAHs on liver function biomarkers strategy by gender.**	**8**
**Table S1 Study Population Characteristics**	**9**
**Table S2 Spearman correlation coefficients among urinary ACs in participants of coking participants.**	**11**
**Table S3 Multivariable linear regression analysis of ACs and HRBs coefficients (β, 95%CI)**	**12**
**Table S4 Estimated posterior inclusion probabilities (PIPs) for HRBs through Bayesian kernel machine regression (BKMR).**	**15**
**Table S5 The mediation effect coefficients of OSBs along with their 95% confidence intervals.**	**15**

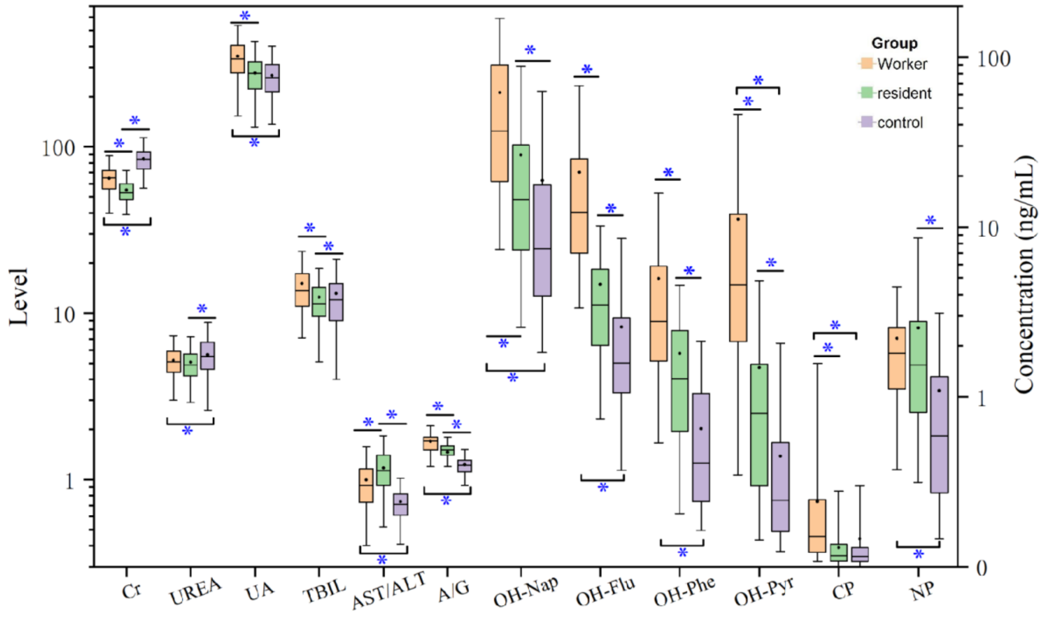

Fig. S1 Level of kidney function and concentrations of urinary pollutants in different regions. The star symbol means significant differences between groups (*: $p < 0.05$).

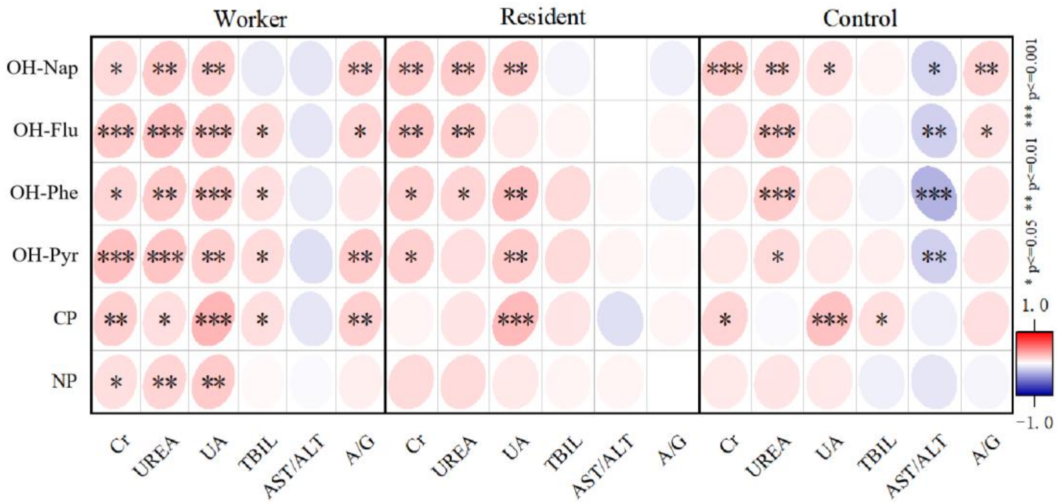

Fig. S2 Spearman correlation between urinary pollutants and kidney function tests. The red-blue color and positive and negative inclination angles of the oval indicate positive and negative correlations,respectively. The star symbol indicate significant Spearman correlations.(*: $p < 0.05$; **: $p < 0.01$; ***: $p < 0.001$)

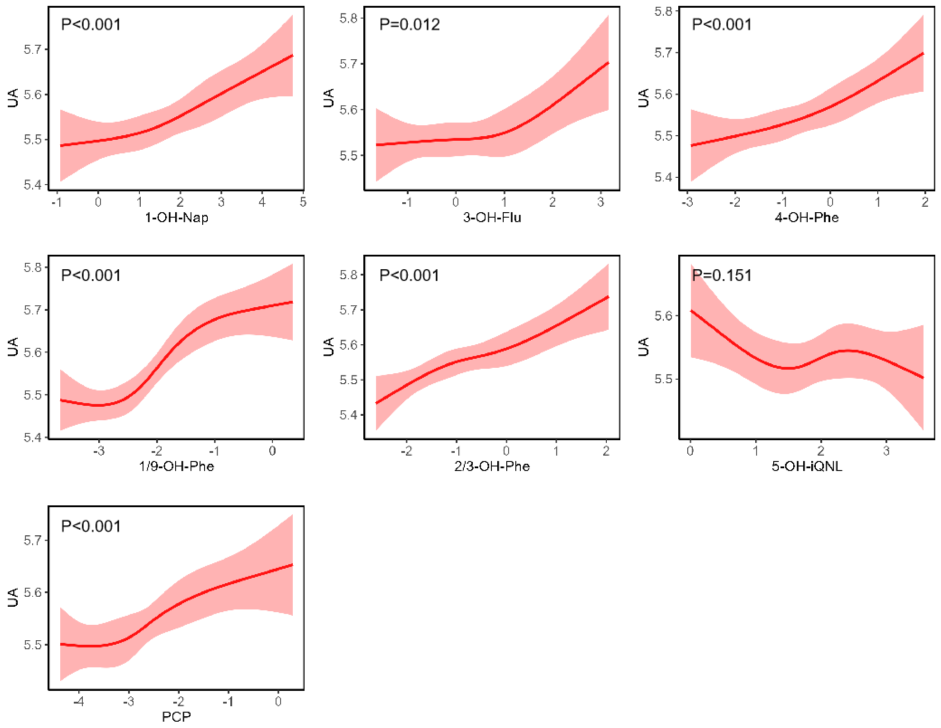

Fig. S3 Adjusted restricted cubic splines of the associations between 1-OH-Nap, 3-OH-Flu, 4-OH-Phe, 1/9-OH-Phe, 2/3-OH-Phe, 5-OH-iQNL, PCP and UA. Models were adjusted for age, gender (categorical) , alcohol consumption (categorical), and smoking (categorical).

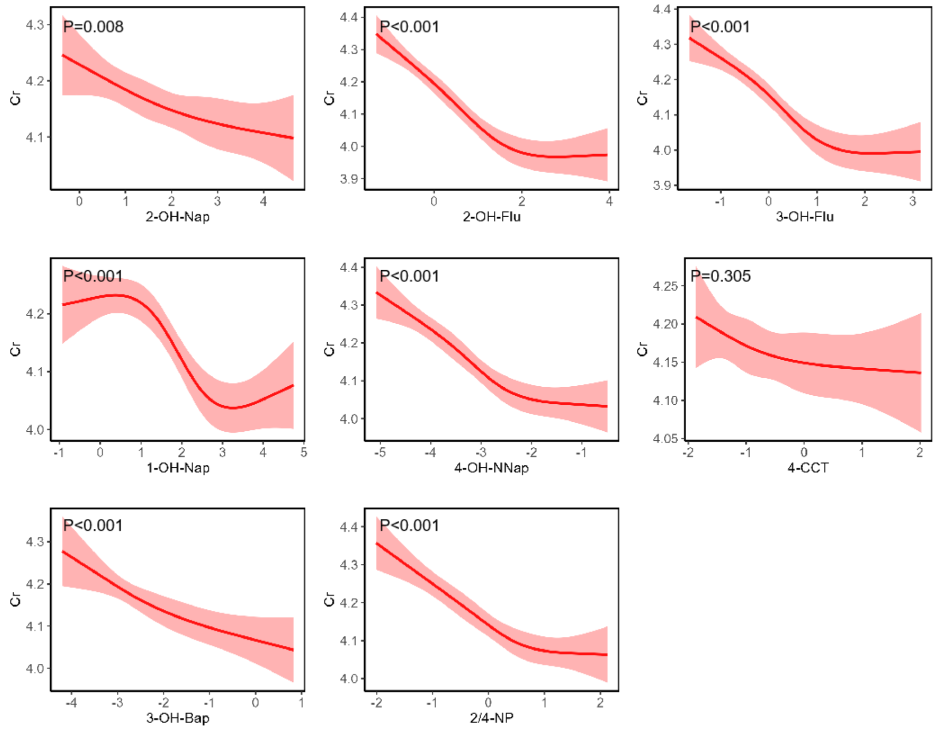

Fig. S4 Adjusted restricted cubic splines of the associations between 1-OH-Nap, 2-OH-Nap, 2-OH-Flu, 3-OH-Flu, 4-OH-NNap, 4-CCT, 3-OH-Bap, 2/4-NP and Cr. Models were adjusted for age, gender (categorical) , alcohol consumption (categorical), and smoking (categorical).

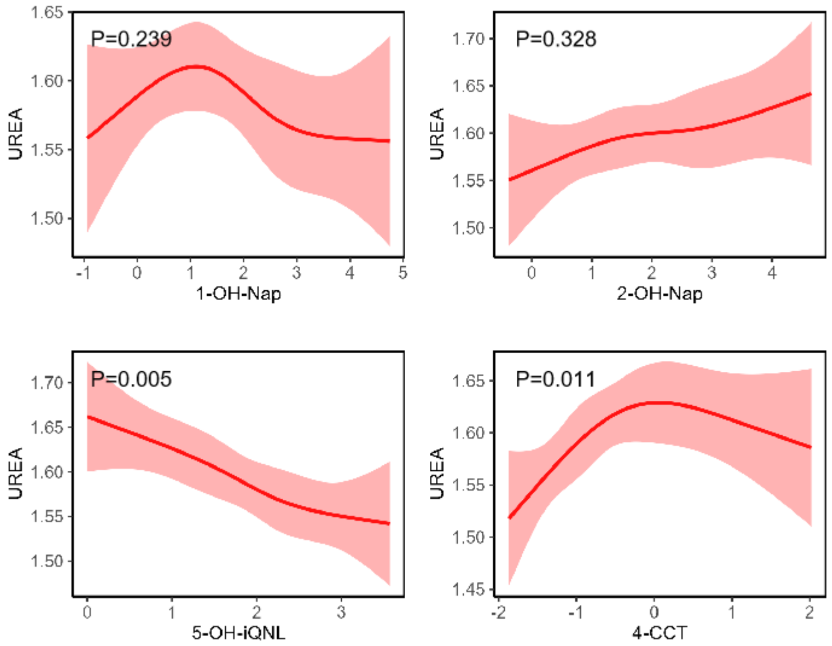

Fig. S5 Adjusted restricted cubic splines of the associations between 1-OH-Nap, 2-OH-Nap, 5-OH-iQNL, 4-CCT and UREA. Models were adjusted for age, gender (categorical), alcohol consumption (categorical), and smoking (categorical).

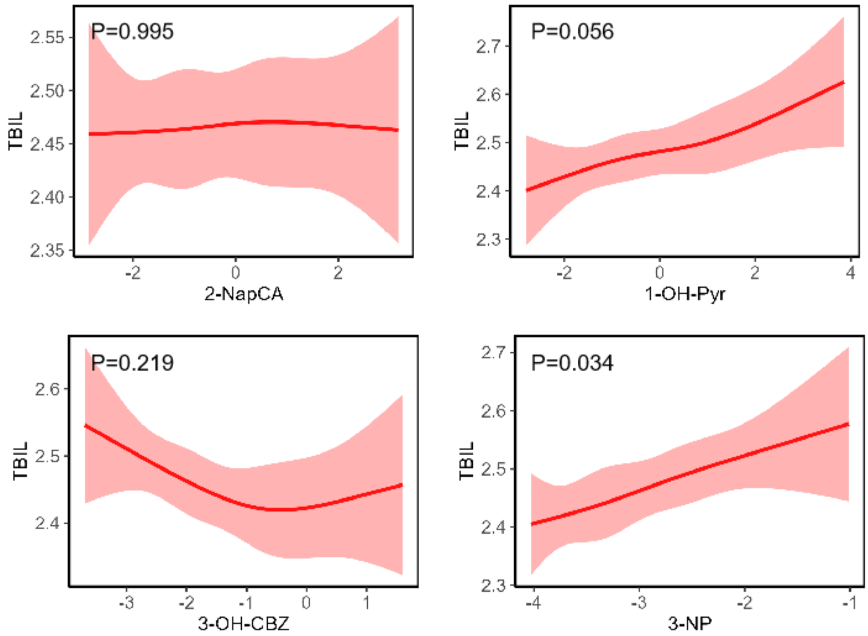

Fig. S6 Adjusted restricted cubic splines of the associations between 1-OH-Pyr, 2-NapCA, 3-OH-CBZ, 3-NP and TBIL. Models were adjusted for age, gender (categorical), alcohol consumption (categorical), and smoking (categorical).

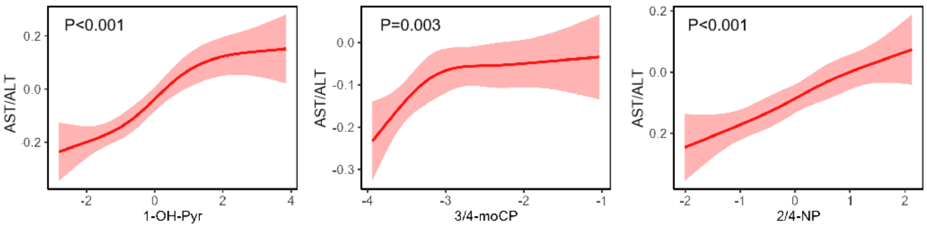

Fig. S7 Adjusted restricted cubic splines of the associations between 1-OH-Pyr, 3/4-moCP, 2/4-NP and AST/ALT. Models were adjusted for age, gender (categorical) , alcohol consumption (categorical), and smoking (categorical).

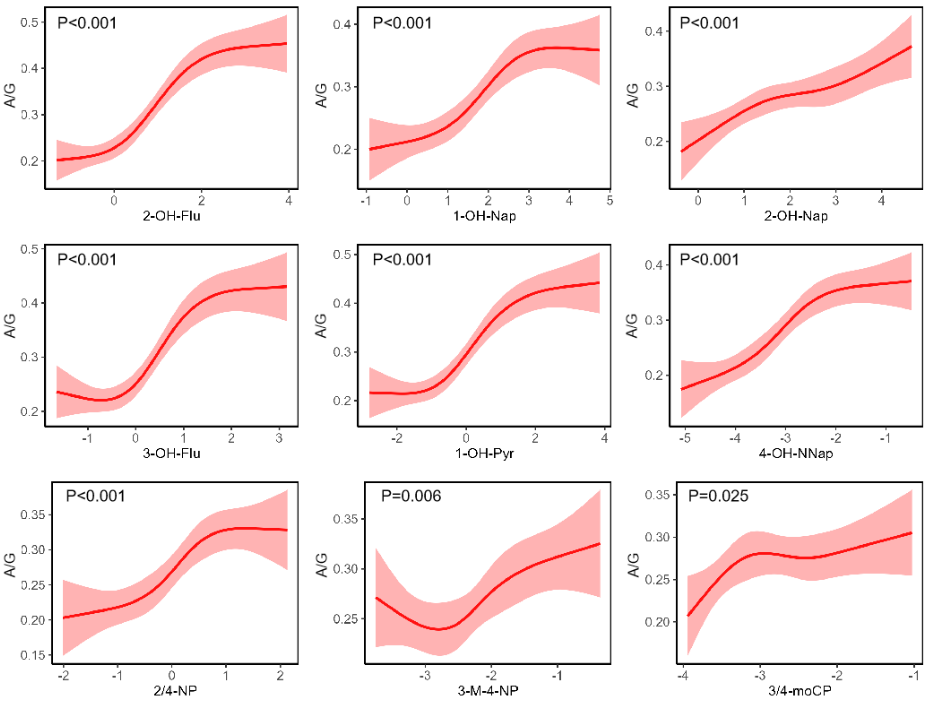

Fig. S8 Adjusted restricted cubic splines of the associations between 1-OH-Nap, 2-OH-Nap, 2-OH-Flu, 1-OH-Pyr, 4-OH-NNap, 2/4-NP, 3-M-4-NP, 3/4-moCP and A/G. Models were adjusted for age, gender (categorical) , alcohol consumption (categorical), and smoking (categorical).

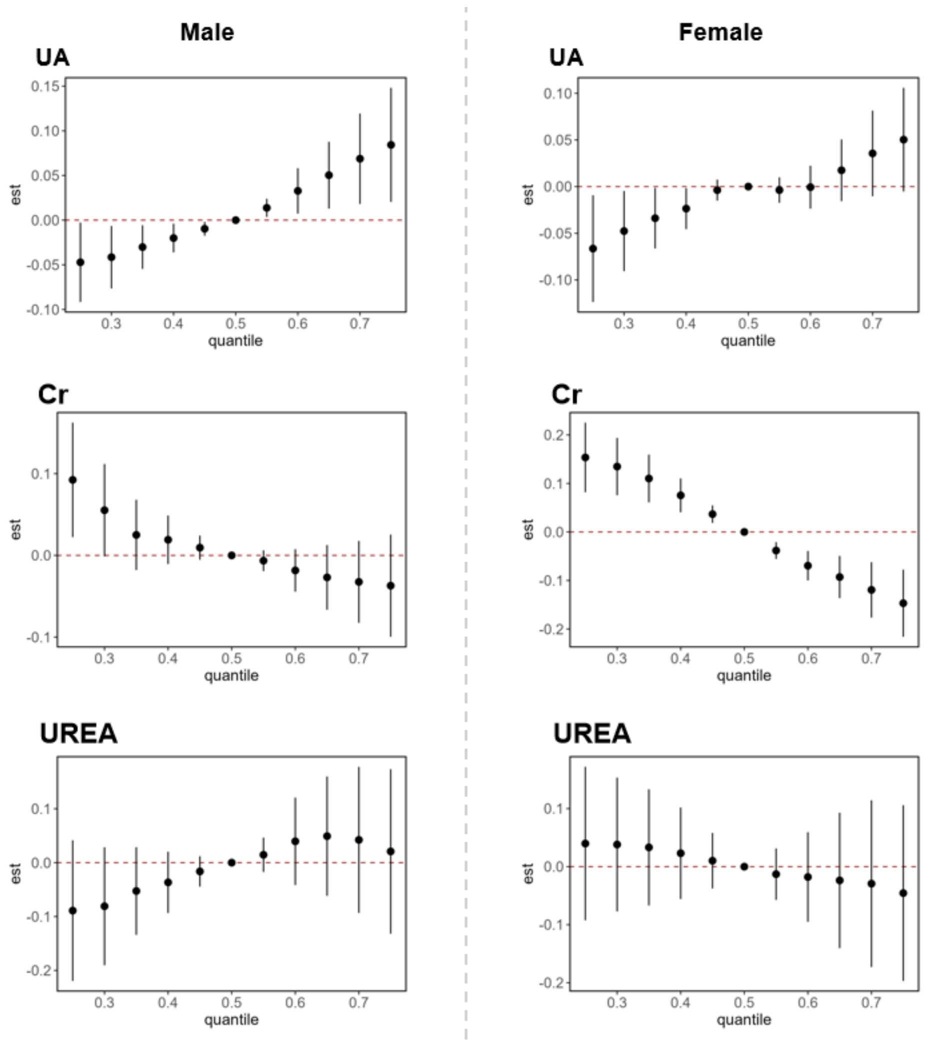

Fig. S9 Joint effects of urinary OH-PAHs on kidney function biomarkers strategy by gender. (UA) Uric acid, (Cr) Creatinine, (UREA) urea. The model adjusted for age (continuous), smoking (categorical) and alcohol use (categorical).

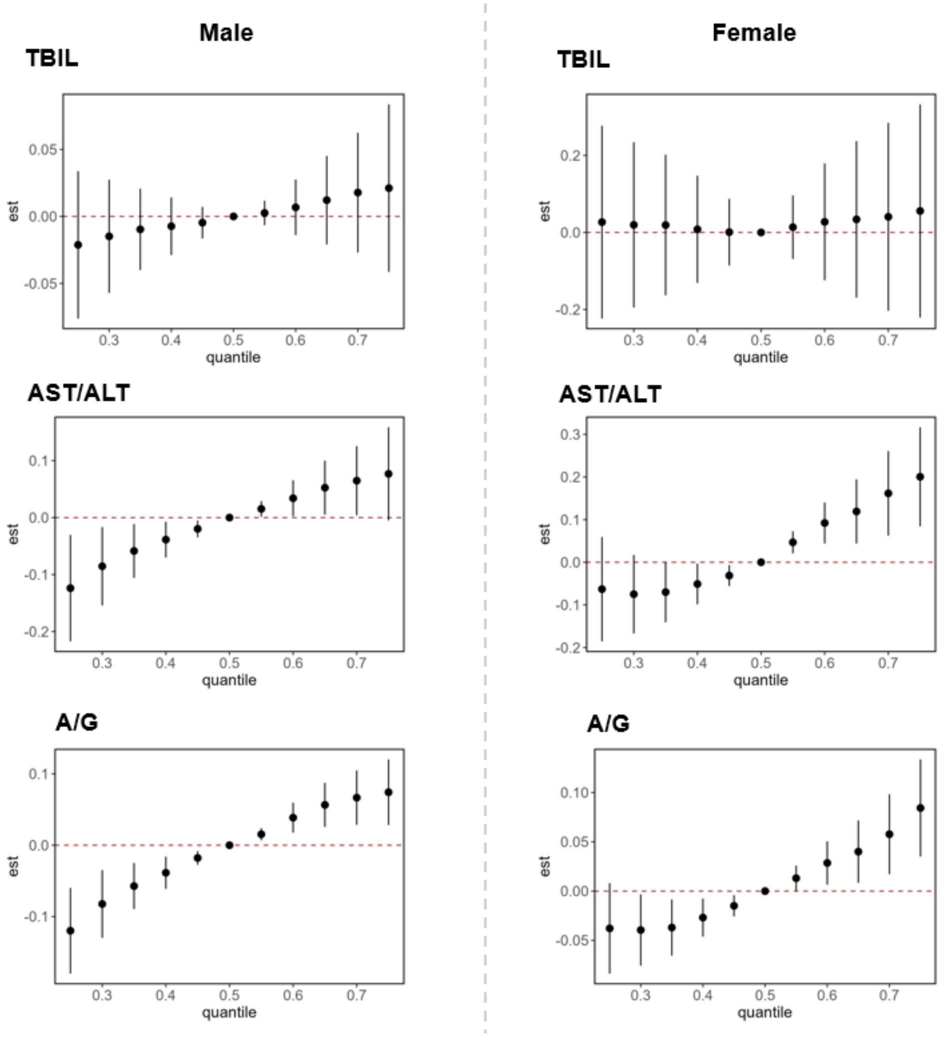

Fig. S10 Joint effects of urinary OH-PAHs on liver function biomarkers strategy by gender. (UA) Uric acid, (Cr) Creatinine, (UREA) urea. The model adjusted for age (continuous), smoking (categorical) and alcohol use (categorical).

Table S1 Study Population Characteristics

Characteristic		Worker (n=226)	Resident (n=163)	Control (n=248)	*p*-Value		
					W-R	W-C	R-C
age		45.88 [35.71, 51.15]	57.38 [49.7, 64.59]	51 [39.25, 58]	**<0.001**	**<0.001**	**<0.001**
sex (%)	Male	174 (77)	46 (28.2)	77 (31)	**<0.001**	**<0.001**	1.000
	Female	52 (23)	117 (71.8)	171 (69)			
Smoking Status (%)	Current smokers	87 (38.5)	125 (76.7)	190 (76.6)	**<0.001**	**<0.001**	1.000
	Other	139 (61.5)	38 (23.3)	58 (23.4)			
Drinking Status (%)	Current drinkers	102 (45.1)	133 (81.6)	219 (88.3)	**<0.001**	**<0.001**	0.415
	Other	124 (54.9)	30 (18.4)	29 (11.7)			
Kidney function biomarkers	Cr (μmol/L)	64.43 [56.78, 72.15]	53.25 [48.09, 60.14]	84.11 [73.37, 93.35]	**<0.001**	**<0.001**	**<0.001**
	UREA (mmol/L)	5.07 [4.43, 5.89]	4.91 [4.19, 5.73]	5.5 [4.6, 6.7]	0.475	**0.003**	**<0.001**
	UA (μmol/L)	337.5 [278, 407.33]	275 [221.75, 324.25]	260.75 [213.73, 311.53]	**<0.001**	**<0.001**	0.778
Liver function biomarkers	TBIL (μmol/L)	15.05 [11 13.6 17.3]	12.48 [9.64, 14.28]	13.14 [9, 15]	**<0.001**	**<0.001**	1.000
	AST/ALT (%)	1 [0.73, 1.16]	1.18 [0.92, 1.4]	0.74 [0.61, 0.82]	**<0.001**	**<0.001**	**<0.001**
	A/G (%)	1.69 [1.53, 1.84]	1.46 [1.35, 1.59]	1.23 [1.11, 1.31]	**<0.001**	**<0.001**	**<0.001**
Urine ACs concentration (ng/mL)	2-OH-Nap	20.16 [8.52, 46.3]	6.97 [3.33, 13.72]	4.34 [2.06, 9.98]	**<0.001**	**<0.001**	**0.020**
	1-OH-Nap	18.53 [8.47, 33.97]	7.39 [3.13, 15.19]	2.6 [1.53, 5.92]	**<0.001**	**<0.001**	**<0.001**
	3-OH-Flu	3.89 [2.39, 8.32]	1.23 [0.74, 2.02]	0.7 [0.46, 1.24]	**<0.001**	**<0.001**	**<0.001**
	2-OH-Flu	8.41 [4.65, 16.78]	2.11 [1.27, 3.74]	0.87 [0.52, 1.75]	**<0.001**	**<0.001**	**<0.001**
	2/3-OH-Phe	1.16 [0.67, 2.59]	0.52 [0.35, 0.9]	0.23 [0.15, 0.4]	**<0.001**	**<0.001**	**<0.001**
	4-OH-Phe	1.31 [0.77, 2.55]	0.63 [0.38, 1.17]	0.32 [0.19, 0.54]	**<0.001**	**<0.001**	**<0.001**
	1/9-OH-Phe	0.27 [0.15, 0.48]	0.11 [0.07, 0.21]	0.07 [0.03, 0.11]	**<0.001**	**<0.001**	**<0.001**
	1-OH-Pyr	4.56 [2.12, 11.9]	0.9 [0.48, 1.55]	0.39 [0.21, 0.73]	**<0.001**	**<0.001**	**<0.001**
	6-OH-Chr	0 [0, 0.03]	0 [0, 0.01]	0 [0, 0]	0.072	**<0.001**	0.146
	3-OH-Bap	0.01 [0, 0.12]	0.01 [0, 0.07]	0 [0, 0.01]	0.750	**0.041**	0.847
	2-NapCA	1.43 [0.65, 3.77]	0.66 [0.26, 2.34]	0.3 [0.13, 0.78]	**<0.001**	**<0.001**	**<0.001**
	4-OH-NNap	0.08 [0.05, 0.15]	0.06 [0.03, 0.1]	0.02 [0.01, 0.04]	**<0.001**	**<0.001**	**<0.001**
	5-OH-iQNL	7.91 [5.62, 12.37]	6.94 [3.53, 12.84]	5.37 [2.94, 9.67]	**0.040**	**<0.001**	**0.011**
	3-OH-CBZ	0.69 [0.36, 1.54]	0.2 [0.11, 0.4]	0.12 [0.06, 0.31]	**<0.001**	**<0.001**	**0.020**
	2-OH-DBF	9.94 [5.78, 18.72]	2.6 [1.67, 5.01]	1.32 [0.81, 2.35]	**<0.001**	**<0.001**	**<0.001**
	4-CCT	0.62 [0.39, 1.05]	0.52 [0.3, 0.83]	0.44 [0.28, 0.81]	**0.004**	**0.004**	0.734

Characteristic		Worker (n=226)	Resident (n=163)	Control (n=248)	*p*-Value		
					W-R	W-C	R-C
	3/4-moCP	0.05 [0.03, 0.09]	0.03 [0.02, 0.05]	0.03 [0, 0.04]	**<0.001**	**<0.001**	0.074
	PCP	0.09 [0.04, 0.28]	0.02 [0.01, 0.08]	0.03 [0, 0.07]	**<0.001**	**<0.001**	1.000
	2/4-NP	1.63 [1, 2.29]	1.39 [0.82, 2.3]	0.68 [0.39, 1.14]	0.546	**<0.001**	**<0.001**
	3-NP	0.05 [0.03, 0.07]	0.03 [0.02, 0.05]	0.02 [0, 0.04]	**<0.001**	**<0.001**	**<0.001**
	3-M-4-NP	0.12 [0.07, 0.21]	0.1 [0.06, 0.23]	0.04 [0, 0.09]	1.000	**<0.001**	**<0.001**
	Total	102 [21.2–1150]	57.0 [11.5–523]	30.7 [7.72–798]	**<0.001**	**<0.001**	**<0.001**
OSBs concentration (ng/mL)	8-OHdG	0.81 [0.54, 1.08]	---	---	---	---	---
	4-OH-NMA	45.26 [11.03, 129.23]	---	---	---	---	---
	8-iso-PGF2α	0.61 [0.46, 0.9]	---	---	---	---	---
	8-iso-15-PGF2α	0.35 [0.24, 0.58]	---	---	---	---	---
	15-PGF2α	1.13 [0.79, 1.63]	---	---	---	---	---

Other, Former smokers and nonsmokers or Former drinkers and nondrinkers; W-R, Comparison between workers and residents; W-C, Comparison between workers and controls; R-C, Comparison between residents and controls; The parameter of median and (and its IQR) was set for non-normal distributed graded data, n (%) for quantal data; The comparison of concentration between the environmental media for different groups was conducted using the Kruskal-Willis method, multiple comparisons are done in pairs by Bonferroni method; ---, The data for this part is unavailable.

Table S2 Spearman correlation coefficients among urinary ACs in participants of coking participants

Group	Variable	Cr	UREA	UA	TBIL	AST/ALT	A/G
worker	OH_Nap	0.148*	0.201**	0.195**	-0.063	-0.085	0.182**
	OH_Flu	0.220***	0.246***	0.218***	0.141*	-0.092	0.169*
	OH_Phe	0.171*	0.204**	0.218***	0.137*	-0.074	0.105
	OH_Pyr	0.260***	0.234***	0.195**	0.153*	-0.113	0.205**
	CP	0.186**	0.134*	0.290***	0.134*	-0.088	0.183**
	NP	0.139*	0.176**	0.216**	0.040	-0.013	0.077
resident	OH_Nap	0.206**	0.204**	0.213**	-0.032	0.020	-0.042
	OH_Flu	0.221**	0.215**	0.092	0.047	0.015	0.046
	OH_Phe	0.191*	0.179*	0.245**	0.149	0.023	-0.040
	OH_Pyr	0.184*	0.135	0.207**	0.149	0.060	0.039
	CP	0.050	0.112	0.277***	0.106	-0.101	0.055
	NP	0.149	0.148	0.098	0.053	0.042	0.010
control	OH_Nap	0.212***	0.167**	0.133*	0.048	-0.154*	0.176**
	OH_Flu	0.122	0.215***	0.071	-0.001	-0.168**	0.137*
	OH_Phe	0.081	0.216***	0.082	-0.021	-0.290***	0.105
	OH_Pyr	0.093	0.146*	0.099	0.074	-0.171**	0.118
	CP	0.160*	-0.016	0.253***	0.133*	-0.056	0.124
	NP	0.080	0.106	0.093	-0.058	-0.084	-0.030

*:Denotes significance in Spearman analysis, where *: $p < 0.05$; **: $p < 0.01$; ***: $p < 0.001$.

Table S3 Multivariable linear regression analysis of ACs and HRBs coefficients (β, 95%CI) (Part 1)

Model	Characteristic	UA	Cr	UREA	TBIL	AST/ALT	A/G
M1	∑ACs*	**0.005(0.003,0.006)**	**-0.008(-0.009,-0.006)**	-0.001(-0.002,0.001)	0.0019(-0.0003,0.0040)	**0.008(0.006,0.010)**	**0.006(0.005,0.007)**
M2	∑ACs*	**0.005(0.003,0.006)**	**-0.008(-0.009,-0.007)**	-0.001(-0.003,0.000)	**0.0026(0.0002,0.0048)**	**0.009(0.006,0.011)**	**0.006(0.005,0.007)**
M3	∑ACs*	**0.004(0.003,0.006)**	**-0.008(-0.009,-0.006)**	-0.001(-0.003,0.000)	0.0021(-0.0001,0.0044)	**0.008(0.006,0.011)**	**0.006(0.004,0.007)**
M1	2-OH-Nap	-0.018(-0.051,0.016)	**0.055(0.028,0.082)**	**0.054(0.025,0.083)**	-0.011(-0.056,0.034)	-0.035(-0.079,0.008)	-0.026(-0.046,-0.005)
M2	2-OH-Nap	-0.018(-0.052,0.015)	**0.054(0.027,0.081)**	**0.051(0.022,0.080)**	-0.004(-0.050,0.041)	-0.031(-0.075,0.012)	-0.029(-0.050,-0.008)
M3	2-OH-Nap	-0.018(-0.052,0.016)	**0.053(0.026,0.080)**	**0.051(0.021,0.080)**	-0.002(-0.048,0.043)	-0.030(-0.074,0.013)	-0.029(-0.049,-0.008)
M1	1-OH-Nap	**0.031(0.000,0.061)**	**-0.037(-0.062,-0.013)**	**-0.047(-0.073,-0.021)**	0.003(-0.038,0.044)	0.036(-0.004,0.075)	**0.019(0.000,0.037)**
M2	1-OH-Nap	**0.031(0.000,0.061)**	**-0.037(-0.062,-0.013)**	**-0.047(-0.073,-0.020)**	0.002(-0.039,0.043)	0.035(-0.004,0.074)	**0.019(0.001,0.038)**
M3	1-OH-Nap	**0.031(0.000,0.061)**	**-0.037(-0.062,-0.013)**	**-0.047(-0.073,-0.020)**	0.003(-0.038,0.044)	0.035(-0.004,0.074)	**0.019(0.001,0.038)**
M1	3-OH-Flu	**-0.111(-0.180,-0.041)**	0.063(0.007,0.119)	0.046(-0.014,0.106)	-0.046(-0.140,0.047)	-0.068(-0.157,0.021)	-0.084(-0.126,-0.041)
M2	3-OH-Flu	**-0.111(-0.180,-0.041)**	0.063(0.008,0.119)	0.046(-0.013,0.106)	-0.047(-0.140,0.046)	-0.069(-0.158,0.021)	-0.083(-0.125,-0.041)
M3	3-OH-Flu	**-0.111(-0.180,-0.041)**	0.063(0.007,0.119)	0.046(-0.013,0.106)	-0.047(-0.139,0.046)	-0.068(-0.158,0.021)	-0.083(-0.125,-0.041)
M1	2-OH-Flu	0.078(-0.005,0.161)	**-0.090(-0.156,-0.023)**	-0.044(-0.115,0.028)	0.007(-0.104,0.119)	0.082(-0.024,0.189)	**0.137(0.086,0.188)**
M2	2-OH-Flu	0.077(-0.006,0.160)	**-0.091(-0.158,-0.024)**	-0.048(-0.120,0.024)	0.017(-0.095,0.128)	0.088(-0.019,0.195)	**0.132(0.081,0.183)**
M3	2-OH-Flu	0.077(-0.006,0.160)	**-0.090(-0.157,-0.023)**	-0.048(-0.119,0.024)	0.015(-0.096,0.127)	0.087(-0.020,0.194)	**0.132(0.081,0.183)**
M1	2/3-OH-Phe	**0.065(0.002,0.127)**	-0.002(-0.053,0.048)	-0.007(-0.061,0.047)	0.066(-0.018,0.151)	-0.049(-0.130,0.031)	-0.022(-0.060,0.016)
M2	2/3-OH-Phe	**0.065(0.002,0.128)**	-0.002(-0.052,0.049)	-0.004(-0.059,0.050)	0.061(-0.024,0.145)	-0.053(-0.134,0.028)	-0.019(-0.057,0.020)
M3	2/3-OH-Phe	**0.065(0.003,0.128)**	-0.002(-0.053,0.048)	-0.005(-0.059,0.050)	0.061(-0.023,0.146)	-0.053(-0.133,0.028)	-0.019(-0.057,0.020)
M1	4-OH-Phe	**-0.049(-0.093,-0.004)**	0.001(-0.034,0.037)	0.027(-0.011,0.065)	-0.028(-0.088,0.032)	-0.021(-0.078,0.037)	-0.011(-0.038,0.017)
M2	4-OH-Phe	**-0.048(-0.093,-0.004)**	0.002(-0.034,0.038)	0.029(-0.010,0.067)	-0.032(-0.091,0.028)	-0.023(-0.080,0.035)	-0.009(-0.036,0.018)
M3	4-OH-Phe	**-0.048(-0.093,-0.003)**	0.001(-0.035,0.036)	0.028(-0.010,0.067)	-0.029(-0.089,0.030)	-0.022(-0.079,0.036)	-0.008(-0.036,0.019)
M1	1/9-OH-Phe	**0.078(0.036,0.121)**	-0.012(-0.046,0.023)	-0.008(-0.045,0.029)	0.010(-0.048,0.068)	-0.027(-0.082,0.028)	-0.008(-0.034,0.018)
M2	1/9-OH-Phe	**0.079(0.036,0.122)**	-0.012(-0.046,0.023)	-0.007(-0.044,0.030)	0.008(-0.050,0.065)	-0.028(-0.083,0.027)	-0.007(-0.033,0.019)
M3	1/9-OH-Phe	**0.078(0.035,0.121)**	-0.008(-0.043,0.026)	-0.006(-0.043,0.031)	0.002(-0.056,0.060)	-0.031(-0.086,0.025)	-0.008(-0.034,0.018)
M1	1-OH-Pyr	-0.001(-0.034,0.032)	-0.015(-0.041,0.011)	-0.025(-0.053,0.004)	**0.044(0.000,0.088)**	**0.052(0.010,0.094)**	**0.022(0.002,0.042)**
M2	1-OH-Pyr	-0.001(-0.034,0.032)	-0.015(-0.041,0.011)	-0.025(-0.053,0.003)	**0.044(0.000,0.088)**	**0.052(0.010,0.094)**	**0.022(0.002,0.042)**
M3	1-OH-Pyr	-0.001(-0.034,0.032)	-0.015(-0.041,0.011)	-0.025(-0.053,0.004)	**0.044(0.000,0.088)**	**0.052(0.010,0.094)**	**0.022(0.002,0.042)**
M1	6-OH-Chr	-0.014(-0.053,0.025)	-0.004(-0.035,0.028)	0.017(-0.017,0.050)	-0.016(-0.068,0.037)	0.023(-0.027,0.073)	-0.017(-0.041,0.006)
M2	6-OH-Chr	-0.015(-0.054,0.024)	-0.004(-0.035,0.028)	0.015(-0.018,0.049)	-0.012(-0.065,0.040)	0.025(-0.025,0.075)	-0.019(-0.043,0.005)
M3	6-OH-Chr	-0.015(-0.054,0.024)	-0.003(-0.035,0.028)	0.015(-0.018,0.049)	-0.013(-0.066,0.039)	0.024(-0.026,0.075)	-0.019(-0.043,0.004)

Table S3 (continued) Multivariable linear regression analysis of ACs and HRBs coefficients (β, 95%CI) (Part 2)

Model	Characteristic	UA	Cr	UREA	TBIL	AST/ALT	A/G
M1	3-OH-Bap	-0.008(-0.032,0.017)	**-0.026(-0.045,-0.006)**	-0.013(-0.034,0.008)	-0.002(-0.034,0.031)	0.024(-0.007,0.055)	0.009(-0.006,0.023)
M2	3-OH-Bap	-0.007(-0.032,0.017)	**-0.026(-0.045,-0.006)**	-0.012(-0.033,0.009)	-0.003(-0.035,0.030)	0.024(-0.007,0.055)	0.009(-0.005,0.024)
M3	3-OH-Bap	-0.007(-0.032,0.017)	**-0.026(-0.045,-0.006)**	-0.012(-0.033,0.009)	-0.003(-0.035,0.029)	0.023(-0.008,0.055)	0.009(-0.005,0.024)
M1	2-NapCA	-0.008(-0.028,0.011)	-0.009(-0.024,0.007)	-0.005(-0.022,0.011)	**-0.045(-0.071,-0.019)**	-0.005(-0.030,0.020)	-0.003(-0.015,0.009)
M2	2-NapCA	-0.008(-0.028,0.011)	-0.009(-0.024,0.007)	-0.006(-0.022,0.011)	**-0.045(-0.071,-0.019)**	-0.005(-0.030,0.020)	-0.003(-0.015,0.009)
M3	2-NapCA	-0.008(-0.028,0.011)	-0.010(-0.026,0.006)	-0.006(-0.023,0.011)	**-0.042(-0.068,-0.016)**	-0.004(-0.029,0.021)	-0.002(-0.014,0.010)
M1	4-OH-NNap	0.022(-0.004,0.048)	**-0.026(-0.047,-0.005)**	0.011(-0.012,0.033)	0.025(-0.010,0.060)	0.020(-0.014,0.053)	**0.030(0.014,0.045)**
M2	4-OH-NNap	0.022(-0.004,0.048)	**-0.026(-0.047,-0.005)**	0.010(-0.013,0.032)	0.027(-0.008,0.062)	0.021(-0.013,0.054)	**0.029(0.013,0.044)**
M3	4-OH-NNap	0.022(-0.004,0.048)	**-0.025(-0.046,-0.004)**	0.010(-0.012,0.033)	0.025(-0.010,0.060)	0.020(-0.013,0.054)	**0.028(0.012,0.044)**
M1	5-OH-iQNL	**-0.036(-0.063,-0.009)**	-0.008(-0.029,0.014)	**-0.027(-0.050,-0.004)**	0.018(-0.018,0.055)	0.011(-0.023,0.046)	-0.008(-0.025,0.008)
M2	5-OH-iQNL	**-0.036(-0.063,-0.009)**	-0.008(-0.029,0.014)	**-0.027(-0.050,-0.003)**	0.017(-0.019,0.053)	0.011(-0.024,0.045)	-0.008(-0.024,0.009)
M3	5-OH-iQNL	**-0.036(-0.063,-0.009)**	-0.008(-0.030,0.013)	**-0.027(-0.050,-0.004)**	0.019(-0.017,0.055)	0.011(-0.023,0.046)	-0.008(-0.024,0.009)
M1	3-OH-CBZ	-0.005(-0.033,0.024)	0.015(-0.008,0.038)	**0.026(0.002,0.051)**	**-0.060(-0.099,-0.022)**	0.014(-0.023,0.051)	0.013(-0.004,0.031)
M2	3-OH-CBZ	-0.005(-0.034,0.024)	0.015(-0.008,0.038)	**0.025(0.000,0.050)**	**-0.058(-0.096,-0.019)**	0.015(-0.021,0.052)	0.012(-0.006,0.029)
M3	3-OH-CBZ	-0.005(-0.034,0.024)	0.014(-0.009,0.037)	**0.025(0.000,0.049)**	**-0.057(-0.095,-0.019)**	0.016(-0.021,0.053)	0.012(-0.006,0.029)
M1	2-OH-DBF	-0.026(-0.072,0.019)	-0.020(-0.056,0.017)	-0.006(-0.045,0.033)	0.043(-0.018,0.104)	0.056(-0.003,0.114)	-0.005(-0.033,0.023)
M2	2-OH-DBF	-0.027(-0.072,0.019)	-0.020(-0.057,0.016)	-0.007(-0.046,0.033)	0.044(-0.017,0.106)	0.057(-0.002,0.115)	-0.006(-0.034,0.022)
M3	2-OH-DBF	-0.027(-0.073,0.018)	-0.017(-0.054,0.019)	-0.005(-0.045,0.034)	0.039(-0.022,0.100)	0.054(-0.004,0.113)	-0.007(-0.035,0.020)
M1	4-CCT	-0.009(-0.037,0.020)	**0.033(0.010,0.055)**	**0.036(0.011,0.060)**	-0.001(-0.039,0.037)	-0.036(-0.073,0.000)	-0.012(-0.029,0.006)
M2	4-CCT	-0.008(-0.037,0.020)	**0.033(0.010,0.055)**	**0.036(0.012,0.060)**	-0.002(-0.040,0.036)	**-0.037(-0.073,-0.001)**	-0.011(-0.029,0.006)
M3	4-CCT	-0.008(-0.037,0.020)	**0.032(0.009,0.054)**	**0.036(0.011,0.060)**	0.000(-0.038,0.038)	-0.036(-0.073,0.000)	-0.011(-0.028,0.006)
M1	3/4-moCP	0.027(-0.003,0.058)	-0.012(-0.037,0.012)	-0.015(-0.042,0.011)	0.012(-0.029,0.054)	**0.053(0.013,0.092)**	**0.024(0.006,0.043)**
M2	3/4-moCP	0.027(-0.004,0.058)	-0.012(-0.037,0.012)	-0.016(-0.042,0.011)	0.013(-0.029,0.054)	**0.053(0.013,0.092)**	**0.024(0.006,0.043)**
M3	3/4-moCP	0.026(-0.005,0.057)	-0.009(-0.034,0.016)	-0.014(-0.041,0.013)	0.006(-0.035,0.048)	**0.050(0.010,0.090)**	**0.023(0.004,0.042)**
M1	PCP	**0.034(0.015,0.054)**	0.004(-0.011,0.020)	-0.006(-0.023,0.011)	0.023(-0.003,0.049)	-0.019(-0.044,0.006)	0.009(-0.003,0.021)
M2	PCP	**0.034(0.015,0.053)**	0.004(-0.011,0.020)	-0.007(-0.023,0.010)	0.024(-0.002,0.050)	-0.018(-0.043,0.007)	0.008(-0.004,0.020)
M3	PCP	**0.034(0.015,0.053)**	0.004(-0.011,0.020)	-0.007(-0.023,0.010)	0.024(-0.002,0.050)	-0.018(-0.043,0.007)	0.008(-0.004,0.020)
M1	2/4-NP	0.024(-0.008,0.057)	**-0.047(-0.073,-0.021)**	0.002(-0.026,0.030)	-0.028(-0.072,0.015)	**0.048(0.006,0.090)**	**0.021(0.001,0.041)**
M2	2/4-NP	0.024(-0.008,0.057)	**-0.048(-0.074,-0.021)**	0.002(-0.026,0.030)	-0.027(-0.071,0.016)	**0.049(0.007,0.090)**	**0.021(0.001,0.040)**
M3	2/4-NP	0.024(-0.008,0.057)	**-0.047(-0.073,-0.021)**	0.002(-0.026,0.030)	-0.027(-0.071,0.016)	**0.049(0.007,0.090)**	**0.020(0.001,0.040)**

Table S3 (continued) Multivariable linear regression analysis of ACs and HRBs coefficients (β, 95%CI) (Part 3)

Model	Characteristic	UA	Cr	UREA	TBIL	AST/ALT	A/G
M1	3-NP	0.013(-0.020,0.045)	0.014(-0.012,0.040)	0.001(-0.027,0.029)	**0.052(0.009,0.096)**	0.016(-0.026,0.058)	0.000(-0.019,0.020)
M2	3-NP	0.013(-0.020,0.045)	0.014(-0.012,0.040)	0.001(-0.027,0.029)	**0.052(0.008,0.095)**	0.016(-0.026,0.058)	0.001(-0.019,0.021)
M3	3-NP	0.013(-0.019,0.046)	0.013(-0.013,0.039)	0.000(-0.028,0.028)	**0.054(0.010,0.097)**	0.017(-0.025,0.059)	0.001(-0.019,0.021)
M1	3-M-4-NP	-0.005(-0.038,0.029)	0.017(-0.010,0.044)	-0.012(-0.041,0.017)	-0.021(-0.066,0.024)	-0.001(-0.044,0.042)	**-0.022(-0.043,-0.002)**
M2	3-M-4-NP	-0.005(-0.038,0.029)	0.017(-0.010,0.044)	-0.011(-0.040,0.018)	-0.024(-0.069,0.021)	-0.003(-0.046,0.040)	**-0.021(-0.041,0.000)**
M3	3-M-4-NP	-0.005(-0.038,0.029)	0.018(-0.009,0.045)	-0.010(-0.039,0.019)	-0.025(-0.070,0.020)	-0.004(-0.047,0.039)	**-0.021(-0.042,-0.001)**

*, multiple linear regression between the total concentration of ACs and different HRBs; The bolded coefficients represent those with p-value < 0.05; M1: model adjusted for gender (categorical) and age (continuous). M2: model adjusted for gender (categorical), age (continuous) and smoking (categorical). M3: model adjusted for gender (categorical), age (continuous), smoking (categorical) and alcohol use (categorical).

Table S4 Estimated posterior inclusion probabilities (PIPs) for liver-kidney functions through Bayesian kernel machine regression.

Variable	Cr	UREA	UA	TBIL	AST/ALT	A/G
2-OH-Nap	1	0.8772	0.534	0.223	0.9044	0.789
1-OH-Nap	0.9494	0.8084	0.866	0.2658	0.7422	0.0328
3-OH-Flu	0.8328	0.4874	0.58	0.4838	0.612	0.2676
2-OH-Flu	0.842	0.5448	0.786	0.468	0.7192	1
2/3-OH-Phe	0.9034	0.5432	0.196	0.6638	0.7192	0.0396
4-OH-Phe	0.8942	0.4524	1	0.3778	0.7454	0.0524
1/9-OH-Phe	0.8732	0.4882	1	0.3194	0.7944	0.0184
1-OH-Pyr	0.8166	0.4916	0.278	0.5116	0.7532	0.6234
6-OH-Chr	0.7714	0.3506	0.604	0.3168	0.6596	0.0062
3-OH-Bap	1	0.4192	0	0.087	0.8168	0.0072
2-NapCA	0.7892	0.498	0.924	0.5104	0.7256	0.032
4-OH-NNap	0.9976	0.483	1	0.2788	1	0.3424
5-OH-iQNL	0.7122	0.6918	0.9	0.156	0.7252	0.0318
3-OH-CBZ	0.8434	0.644	0.55	0.6778	0.7766	0.0216
2-OH-DBF	1	0.5086	0.468	0.8882	0.9676	0.014
4-CCT	0.903	0.5026	0.124	0.3586	0.75	0.0978
3/4-moCP	0.428	0.6042	0.652	0.6178	0.6708	0.7526
PCP	0.802	0.3704	0.69	0.9814	0.7666	0.0102
2/4-NP	0.8982	0.445	0.958	0.3576	0.7172	0.0242
3-NP	0.7334	0.447	0.412	0.887	0.729	0.01
3-M-4-NP	0.7714	0.4592	0.828	0.2756	0.7132	0.0068

Table S5 The mediation effect coefficients of OSBs along with their 95% confidence intervals.

ACs	OSBs	Indirect(ab)	Direct(c')	Total(c)
3-OH-Flu	8-OHdG	0.032(0.002,0.077)	0.093(-0.051,0.241)	0.125(-0.011,0.269)
2-OH-Flu	8-OHdG	0.033(0.002,0.077)	0.094(-0.058,0.237)	0.127(-0.013,0.268)
2/3-OH-Phe	8-OHdG	0.03(0.003,0.067)	0.089(-0.062,0.235)	0.119(-0.038,0.261)
4-OH-Phe	8-OHdG	0.037(0.005,0.086)	0.064(-0.089,0.211)	0.102(-0.037,0.242)
1/9-OH-Phe	8-OHdG	0.036(0.004,0.083)	0.09(-0.076,0.198)	0.126(-0.037,0.226)
1-OH-Pyr	8-OHdG	0.028(0.002,0.070)	0.093(-0.081,0.273)	0.121(-0.044,0.291)
3-OH-CBZ	8-OHdG	0.034(0.005,0.071)	0.025(-0.126,0.164)	0.058(-0.093,0.196)
2/4-NP	8-OHdG	0.021(0.004,0.081)	-0.001(-0.164,0.105)	0.02(-0.120,0.129)
3-M-4-NP	8-OHdG	0.022(0.004,0.130)	-0.052(-0.273,0.116)	-0.031(-0.197,0.174)
2-OH-Nap	8-iso-PGF2a	0.029(0.001,0.085)	-0.036(-0.153,0.087)	-0.007(-0.120,0.134)
4-OH-NNap	8-iso-PGF2a	0.007(0.000,0.212)	0.047(-0.648,0.107)	0.054(-0.525,0.247)
2-OH-Nap	8-iso-15-PGF2a	0.035(0.002,0.101)	-0.042(-0.167,0.082)	-0.007(-0.120,0.134)
3-M-4-NP	8-iso-15-PGF2a	0.025(0.003,0.143)	-0.056(-0.270,0.085)	-0.031(-0.197,0.174)
